# Synthesis and *In Vitro* Cytotoxic Activity of Novel Chalcone-Like Agents

**Published:** 2013-11

**Authors:** Bahram Letafat, Raheleh Shakeri, Saeed Emami, Saeedeh Noushini, Negar Mohammadhosseini, Nayyereh Shirkavand, Sussan Kabudanian Ardestani, Maliheh Safavi, Marjaneh Samadizadeh, Aida Letafat, Abbas Shaﬁee, Alireza Foroumadi

**Affiliations:** 1 Department of Chemistry, Central Tehran-Branch, Islamic Azad University, Tehran, Iran; 2 Institute of Biochemistry and Biophysics, University of Tehran, Tehran, Iran; 3 Department of Medicinal Chemistry and Pharmaceutical Sciences Research Center, Faculty of Pharmacy, Mazandaran University of Medical Sciences, Sari, Iran; 4 Department of Medicinal Chemistry, Faculty of Pharmacy and Pharmaceutical Sciences Research Center, Tehran University of Medical Sciences, Tehran, Iran; 5 Department of Chemistry, Tabriz Branch, Islamic Azad University, Tabriz, Iran

**Keywords:** Chalcones, Cytotoxic activity, 4-Chromanone, Synthesis

## Abstract

***Objective(s):*** Chalcones and their rigid analogues represent an important class of small molecules having anticancer activities. Therefore, in this study the synthesis and cytotoxic activity of new 3-benzylidenchroman-4-ones were described as rigid chalcone analogues.

***Materials and Methods:*** The reaction of resorcinol with 3-chloropropionic acid in the presence of CF_3_SO_3_H was afforded corresponding propiophenone. It was cyclized using 2M NaOH to give 7-hydroxy-4-chromanone. *O*-Alkylation of 7-hydroxy-4-chromanone with alkyl iodide in the presence of K_2_CO_3_ gave 7-alkoxychroman-4-one. Finally, condensation of chroman-4-one derivatives with different aldehydes afforded target compounds in good yields. The newly synthesized compounds were tested *in vitro* against different human cancer cell lines including K562 (human erythroleukemia), MDA-MB-231 (human breast cancer), and SK-N-MC (human neuroblastoma) cells. The cell viability was evaluated using MTT colorimetric assay.

***Results:*** Most of the compounds showed good inhibitory activity against cancer cells. Among them, compound **4a** containing 7-hydroxy group on chromanone ring and 3-bromo-4-hydroxy-5-methoxy substitution pattern on benzylidene moiety was the most potent compound with IC_50_ values ≤ 3.86 µg/ml. It was 6-17 times more potent than etoposide against tested cell lines.

***Conclusion:*** We described synthesis and cytotoxic activity of poly-functionalized 3-benzylidenechroman-4-ones as new chalcone-like agents. These compounds can be considered as conformationally constrained congeners of chalcones to tolerate the poly-functionalization on the core structures for further optimization.

## Introduction

Despite significant advances in medical sciences, cancer is still an intractable disease in humans and is a major cause of death around the world. Considering the existing cancer therapies, chemotherapy is one of the most important treatments in the cancer management (1). Cancer is uncontrolled growth of abnormal cells in the body and ideal chemotherapeutic agents are selective inhibitors of the proliferation of only abnormal cells with least or no effect on normal cells. However, the available anticancer agents often act on cells with rapid metabolism and proliferation, and cannot distinguish between cancer and normal cells. Therefore, identification of novel potent, selective, and less toxic anticancer agents remains one of the most active area in the field of medicinal chemistry and drug design (2). On the other hand, development of resistance against the existing anticancer drugs keeps research window open in the search of newer small molecule chemotherapeutics (3).

**Figure 1:**
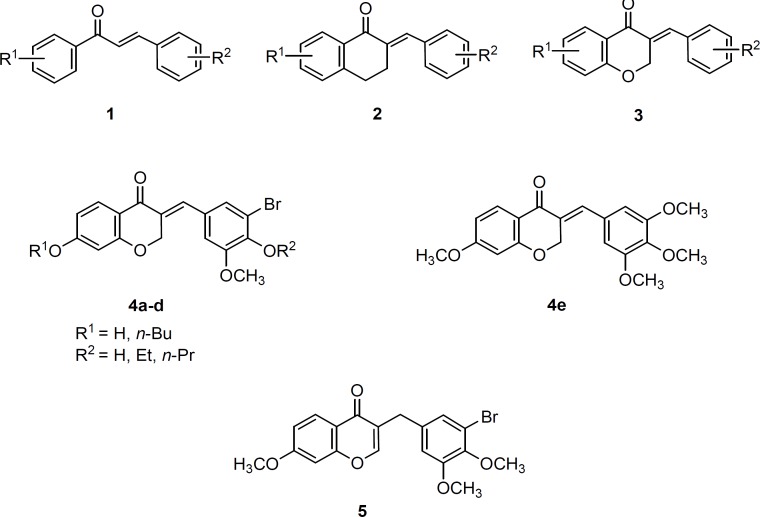
Chalcones **1** and chalcone-like compounds **2 **and** 3** represent an important class of small molecules useful as cytotoxic agents. Structure of target compounds **4a-e** and **5** were designed as new cytotoxic agents

Among the current identified anticancer agents, chalcones **1** and chalcone-like (chalconoid) compounds represent an important class of small useful molecules in cancer chemotherapy. Chalcone template consists of two aromatic rings linked by a three-carbon enone fragment (Figure 1). The double bond of the enone system is essential for anticancer activity of chalcone prototypes (4). The advantage of chalcones is the low propensity to interact with DNA and to decrease the risk of mutagenicity as a common side effect of current chemotherapeutic agents (5). 

A literature survey revealed that the structural modifications of the chalcones mostly focused on the replacement of the phenyl rings with heterocyclic rings and poly-aromatic groups (6, 7), introduction of different substituents on the phenyl moieties (8, 9) and cyclization of the chalcone to give rigid analogs (10). As rigid analogs of chalcones, a series of 2-benzylidene-1-tetralones (**2**, Figure 1) were reported to exhibit promising cytotoxic activities against human Molt 4/C8 leukemia, CEM lymphoma and murine L1210 lymphoma cells (11, 12). Subsequently, 3-benzylidenechroman-4-ones (**3**, Figure. 1) in which the 4-methylene group of the 2-benzylidene-1-tetralones was replaced by an oxygen atom was synthesized and assessed for cytotoxic activity by Perjési *et al* (13). Their study revealed that a number of 3-benzylidenechroman-4-ones **3** exhibited greater cytotoxic activity than the corresponding 2-benzylidene-1-tetralones **2**. We therefore designed poly-functionalized 3-benzylidenechroman-4-ones** 4** as new potential anticancer agents. Thus, in continuation of research program to find a novel anticancer drug (14-16) we describe here, the synthesis and cytotoxic activity of poly-substituted 3-(benzylidene)-4-chromanones **4a-e** and related benzylchromenone analogue **5** (Figure 1).

## Materials and Methods

***Chemistry***

**Scheme 1:**
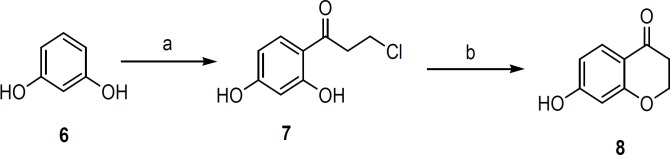
Synthesis of key intermediate **8**.Reagents and* conditions:* (a) 3-chloropropionic acid, CF_3_SO_3_H (3 equiv), 80°C, 30 min; (b) 2.0 M NaOH

**Scheme 2:**
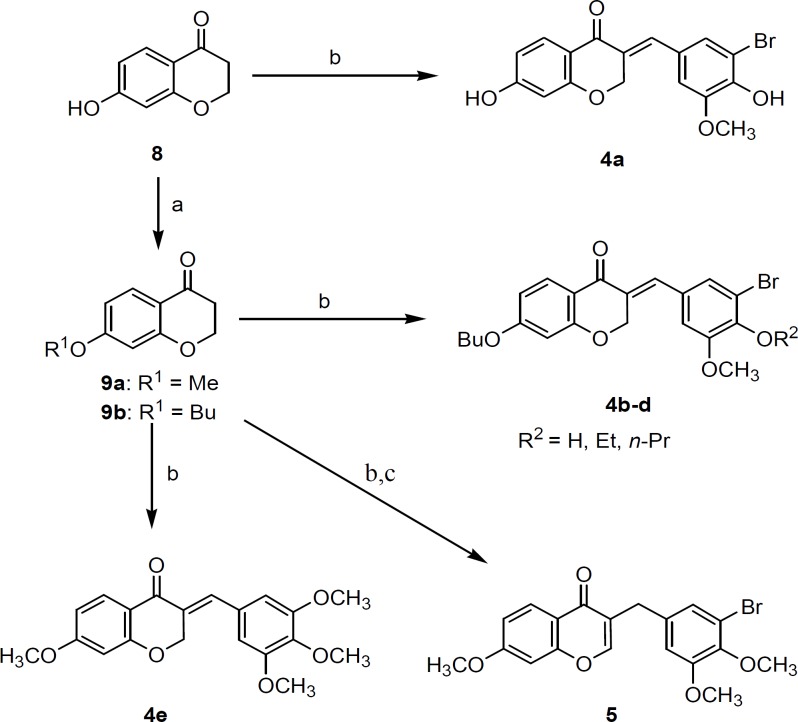
Synthesis of designed compounds **4a-e** and **5**. *Reagents and conditions*: (a) appropriate alkyl iodide, K_2_CO_3_, DMF; (b) appropriate aldehyde, HCl, EtOH; (c) rhodium (III) chloride, H_2_O

All chemical reagents and solvents were purchased from Merck AG (Darmstadt, Germany). The target compounds were synthesized as outlined in Schemes 1 and 2. 7-Alkoxychroman-4-ones **9a,b** and poly-substituted benzaldehydes were prepared as literature methods (17, 18). Melting points were determined using Kofler hot stage apparatus and are uncorrected. The IR spectra were obtained on a Shimadzu 470 spectrophotometer (potassium bromide disks). NMR spectra were recorded using a Bruker 400 MHz spectrometer (Bruker Bioscience, Billerica, MA, USA) and chemical shifts are expressed as δ (ppm) with tetramethylsilane (TMS) as internal standard. 

*General procedure for the synthesis of poly-substituted 3-(benzylidene)-4-chromanones* ***4a-e*** A solution of 7-substituted chroman-4-one (0.5 mmol) (19), substituted aldehyde (0.5 mmol) and HCl (1 ml) in ethanol (5 ml) was heated under reﬂux for 6 hr. The mixture was concentrated under reduced pressure and diluted with water (20 ml). The aqueous solution was extracted with ethyl acetate (3×30 ml) and the organic phase was concentrated under reduced pressure. The residue was crystallized from ethanol/chloroform to give compounds **4a–e**.

3*-(3-Bromo-4-hydroxy-5-methoxybenzylidene)-7-hydroxychroman-4-one* *(****4a****)*

Yield: 38%; mp: 122-124C; IR (KBr, cm^-1^) ν_max_: 1670 (C=O).^ 1^H-NMR (CDCl_3_) δ: 3.88 (s, 3H, OCH_3_), 5.39 (d, 2H, H_2_-Chroman, *J* = 1.4 Hz), 6.32 (d, 1H, H_8_-Chroman, *J* = 2.2 Hz), 6.56 (dd, 1H, H_6_-Chroman, *J* = 8.6 and 2.2 Hz), 7.04 (s, 1H, Ar), 7.19 (s, 1H, Ar), 7.58 (s, 1H, =CH), 7.74 (d, 1H, H_5_-Chroman, *J* = 8.6 Hz), 10.11 (s, 1H, OH), 10.68 (s, 1H, OH). MS (m/z, %): 378 ([M+2]^+^, 100), 376 (M^+^, 100), 361 (89), 359 (90), 344 (63), 297 (43).

*3-(3-Bromo-4-* *hydroxy-5-* *methoxybenz* *ylidene* *)-7-butoxychroman-4-one (* ***4b*** *)*

Yield: 69%; m.p.: 134-136C; IR (KBr, cm^-1^) ν_max_: 1606 (C=O); ^1^H-NMR (CDCl_3_) δ: 0.99 (t, 3H, CH_3_, *J* = 7.3Hz), 1.52 (sextet, 2H, CH_2_, *J* = 7.5 Hz), 1.79 (sextet, 2H, CH_2, _*J* = 6.5 Hz), 3.94 (s, 3H, OCH_3_), 4.01 (t, 2H, OCH_2_, *J* = 6.5 Hz), 5.34 (d, 2H, H_2_-Chroman, *J* = 2.3 Hz), 6.22 (s, 1H, OH), 6.39 (d, 1H, H_8_-Chroman, *J* = 2.3 Hz), 6.64 (dd, 1H, H_6_-Chroman_, _*J* = 6.4 and 2.4 Hz)_, _6.77-6.79 (m, 1H, Ar), 7.04-7.07 (m, 1H, Ar), 7.70 (s, 1H, =CH), 7.95 (d, 1H, H_5_-Chroman, *J* = 8.8 Hz). MS (m/z, %):434 ([M+2]^+^, 100), 432 (M^+^, 100), 415 (66), 402 (77), 400 (78), 354 (55).

*3-(3-Bromo-4-ethoxy-5-methoxybenzylidene)-7-butoxychroman-4-one (* ***4c*** *)*

Yield: 32%; m.p.: 72-74C; IR (KBr, cm^-1^) ν_max_: 1612 (C=O); ^1^H-NMR (CDCl_3_) δ: 0.92-1.04 (m, 3H, CH_3_), 1.43 (t, 3H, CH_3_, *J* = 7.0 Hz), 1.43-1.46 (m, 2H, CH_2_), 1.76-1.83 (m, 2H, CH_2_), 3.89 (s, 3H, OCH_3_), 3.99 (t, 2H, OCH_2_, *J* = 6.5 Hz), 4.13 (q, 2H, OCH_2_, *J* = 7.0 Hz), 5.33 (d, 2H, H_2_-Chroman, *J* = 1.8 Hz), 6.40 (d, 1H, H_8_-Chroman, *J* = 2.3 Hz), 6.64 (dd, 1H, H_6_-Chroman_, _*J* = 6.4 and 2.4 Hz)_, _6.79-6.81 (m, 1H, Ar), 7.04-7.07 (m,1H, Ar), 7.72 (s, 1H, =CH), 7.95 (d, 1H, H_5_-Chroman, *J* = 8.8 Hz). MS (m/z, %):462 ([M+2]^+^, 88), 460 (M^+^, 90), 447 (98), 445 (100), 398 (78), 383 (55).

*3-(3-Bromo-5-methoxy-4-* *propoxybenzylidene)-7-butoxychroman-4-one (* ***4d*** *)*

Yield: 92%; m.p.: 59-61C; IR (KBr, cm^-1^) ν_max_: 1612 (C=O); ^1^H-NMR (CDCl_3_) δ: 0.99 (t, 3H, CH_3_, *J* = 7.3 Hz), 1.08 (t, 3H, CH_3_, *J* = 7.4 Hz), 1.79 (sextet, 2H, CH_2_, *J* = 7.3 Hz), 1.85 (sextet, 2H, CH_2, _*J* = 7.4 Hz), 3.88 (s, 2H, CH_2_), 3.99-4.04 (m, 4H, 2CH_2_O), 5.32 (d, 2H, H_2_-Chroman, *J* = 1.8 Hz), 6.39 (d, 1H, H_8_-Chroman, *J* = 2.3 Hz), 6.64 (dd, 1H, H_6_-Chroman_, _*J* = 6.4 and 2.3 Hz)_, _6.79-6.81 (m, 1H, Ar), 7.04-7.06 (m, 1H, Ar), 7.70 (s, 1H, =CH), 7.95 (d, 1H, H_5_-Chroman, *J* = 8.8 Hz). MS (m/z, %): 476 ([M+2]^+^, 70), 474 (M^+^, 69), 461 (100), 459 (99), 416 (78), 397 (46).

*3-(3,4,5-Trimethoxybenzylidene)-7-methoxychroman-4-one (* ***4e*** *)*

Oily substance; Yield: 13%; IR (KBr, cm^-1^) ν_max_: 1669 (C=O). ^1^H-NMR (CDCl_3_) δ: 3.83 (s, 3H, OCH_3_), 3.86 (s, 3H, OCH_3_), 3.90(s, 3H, OCH_3_), 3.92 (s, 3H, OCH_3_), 5.37 (d, 2H, H_2_-Chroman, *J* = 1.8 Hz), 6.40 (d, 1H, H_8_-Chroman, *J* =2.3 Hz), 6.64 (dd, 1H, H_6_-Chroman, *J* = 8.8 and 2.2 Hz), 7.13 (s, 2H, Ar), 7.77 (s, 1H, =CH), 7.95 (d, 1H, H_5_-Chroman, *J* = 8.8 Hz). MS (m/z, %):356 (M^+^, 90), 341 (100), 326 (85), 312 (44), 281 (65).

*Preparation of 3-(3-bromo-4,5-dimethoxybenzyl)-7-methoxy-4H-chromen-4-one (* ***5*** *)*

A solution of 7-methoxychroman-4-one (0.5 mmol), 3-bromo-4,5-dimethoxybenzaldehyde (0.5 mmol) and HCl (1 ml) in ethanol (5 ml) was reﬂuxed for 6 hr. After completion of the reaction, the mixture was concentrated under reduced pressure and diluted with water (20 ml). The aqueous solution was extracted with ethyl acetate (3×30 ml) and the organic phase was concentrated under reduced pressure. The residue was mixed with rhodium (III) chloride (20 mg), and EtOH-H_2_O (80:20, 2 ml) and the mixture was refluxed for 20 min. After completion of the reaction, the mixture was concentrated under reduced pressure and the residue was purified using a short silica gel column to give compound **5**. Oily substance; Yield: 70%; IR (KBr, cm^-1^) ν_max_: 1668 (C=O).^ 1^H-NMR (CDCl_3_) δ: 3.87 (s, 3H, OCH_3_), 3.91 (s, 3H, OCH_3_), 3.92 (s, 3H, OCH_3_), 5.33 (d, 2H, CH_2_benzylic, *J* = 1.9Hz), 6.42 (d, 1H, H_8_-Chroman, *J* = 2.4 Hz), 6.66 (dd, 1H, H_6_-Chroman, *J* = 8.8 and 2.4 Hz), 6.81 (s, 1H, Ar), 7.06 (s, 1H, Ar), 7.72 (s, 1H, =CH chromene), 7.97 (d, 1H, H_5_-Chroman, *J*= 8.8 Hz). MS (m/z,%):406 ([M+2]^+^, 74), 404 (M^+^, 76), 391 (100), 389 (100), 369 (77), 326 (56).

***Cytotoxicity assay***

The *in vitro* cytotoxic activity of each synthesized compounds **4a-e **and** 5** was evaluated against K562 (human erythroleukemia), MDA-MB-231 (humanbreast cancer), and SK-N-MC (human neuroblastoma) cells using MTT colorimetric assay according to the previously published protocol (20). Cancer cells were grown in RPMI-1640 medium supplemented with 10% heat-inactivated fetal calf serum (Gibco BRL), 100 μg/ml streptomycin and 100 U/ml penicillin at 37°C in a humidified atmosphere with 5% CO_2_ in air.

Briefly, each cell line in log-phase of growth was harvested by trypsinization, resuspended in complete growth medium to give a total cell count of 5×10^4^ cells/ml. Then, 195 µl of the cell suspension was seeded into the wells of 96-well plates (Nunc, Denmark). The plates were incubated overnight in a humidified air atmosphere at 37°C with 5% CO_2_. After overnight incubation, 5 µl of the media containing various concentrations of the compounds was added per well in triplicate (final concentration 1, 5, 10 and 20 µg/ml). The plates were incubated for further 24 hr. The final concentration of DMSO in the highest concentration of the applied compounds was 0.1%. In each plate, there were three control wells (cells without test compounds) and three blank wells (the medium with 0.1% DMSO) for cell viability. Etoposide was used as positive control for cytotoxicity. After treatment, the medium was removed and 200 µl phenol red-free medium containing MTT (1 mg/ml), was added to wells, followed by 4 hr of incubation. After incubation, the culture medium was then replaced with 100 µl of DMSO and the absorbance of each well was measured by using a microplate reader at 570 nm. For each compound, the concentration causing 50% cell growth inhibition (IC_50_) were compared to the control was calculated from concentration-response curves by regression analysis.

***Acridine orange/ethidium bromide staining method***

MDA-MB-231 cells were treated with IC_50_ concentration of compound **4a**, etoposide as positive control and 1% DMSO as negative control for 24 hr. Cells were washed with PBS and one microlitre of acridine orange/ethidium bromide mixture (100 µg/ml AO and 100 µg/ml EB in PBS) was mixed with 9 μl of cell suspension on a clean microscope slide. The suspension was immediately examined by fluorescence microscopy. Acridine orange is taken up by both living and dead cells and emits green fluorescence as a result of intercalation in double-stranded DNA. Ethidium bromide is taken up only by dead cells and emits red fluorescence by intercalation into DNA.

## Results

***Chemistry***

The key intermediate 7-hydroxychroman-4-one (8) was prepared as illustrated in Scheme 1 by using reported method (17). The reaction of resorcinol (6) with 3-chloropropionic acid in the presence of trifluoromethane sulfonic acid gave 3-chloro-1-(2,4-dihydroxyphenyl)propan-1-one (7) which was cyclized using 2M NaOH to give 7-hydroxy-4-chromanone (8). Compound **8 **was alkylated by iodoalkane in the presence of potassium carbonate in DMF to furnish 7-alkoxychroman-4-one **9a,b**. Condensation of compound **8** or **9a,b** with suitable aldehydes in the presence of HCl gave the corresponding target compounds **4a-e**. Condensation of 7-methoxychroman-4-one with 3-bromo-4,5-dimethoxybenzaldehyde and subsequent treatment with rhodium (III) chloride and EtOH-H_2_O gave the 3-benzylchromene-4-one derivative **5** (Scheme 2).

**Table 1:** Cytotoxic activity (IC_50_, µg/ml) of compounds **4a-e** and **5** against different cell lines in comparison with etoposide

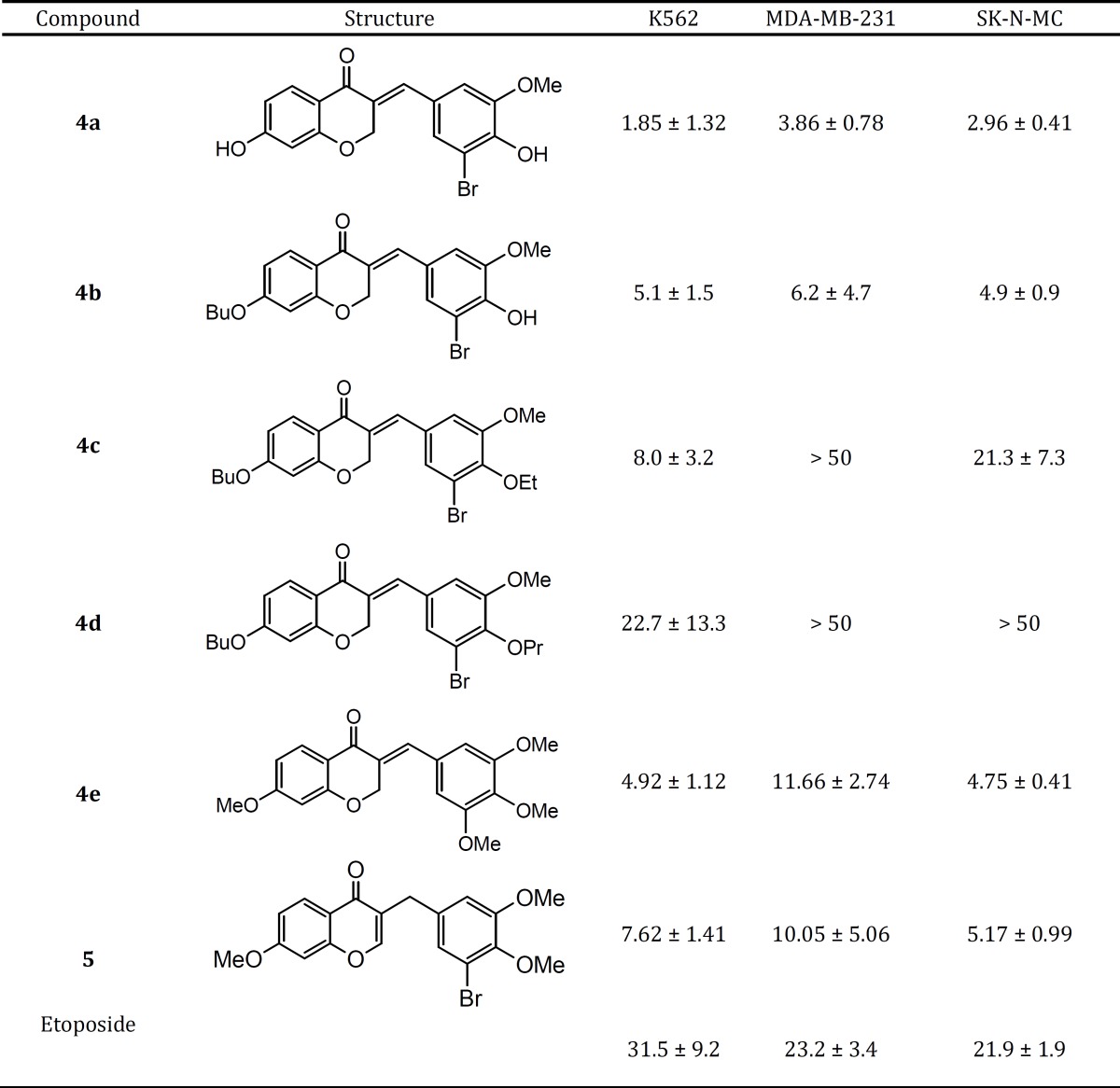

***Cytotoxic activity***

**Figure 2:**
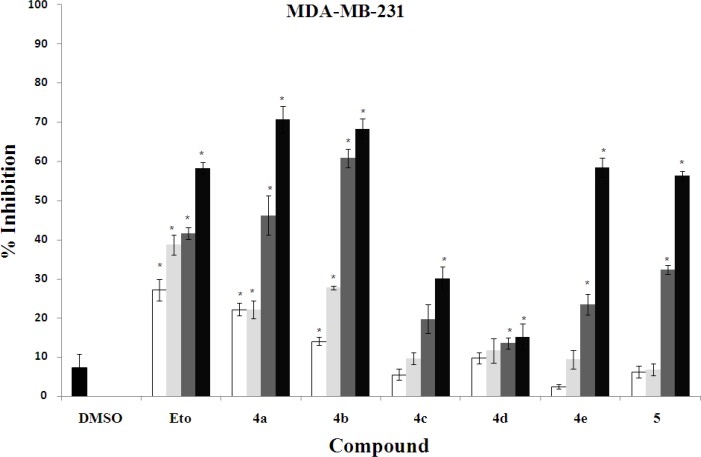
Cytotoxic effects of compounds on MDA-MB-231 cell line. Cell viability assays using MDA-MB-231 cells treated with increasing doses of etoposide (10, 15, 20 and 30 µg/ml) and synthesized compounds (0.5, 1, 5, and 10 µg/ml). *P<0.05 compared to DMSO control-treated MDA-MB-231 cells

**Figure 3:**
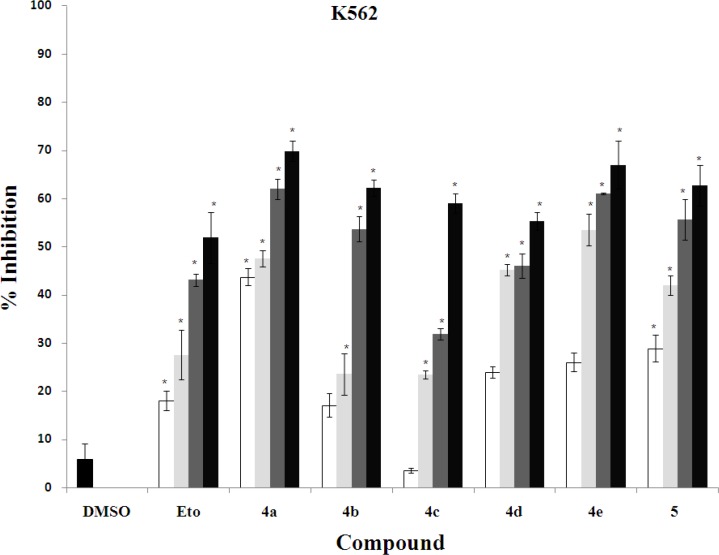
Cytotoxic effects of compounds on K562 cell line. Cell viability assays using K562 cells treated with increasing doses of etoposide (10, 15, 20 and 30 µg/ml) and synthesized compounds (0.5, 1, 5, and 10 µg/ml). *P<0.05 compared to DMSO control-treated K562 cells

**Figure 4:**
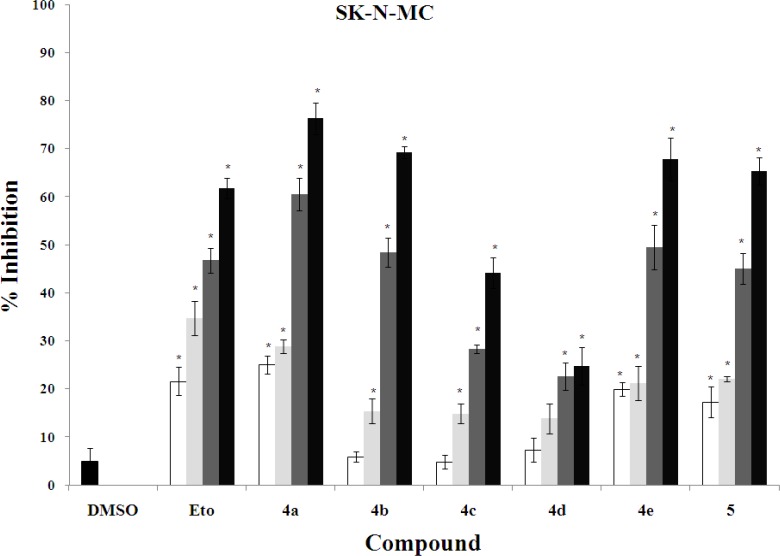
Cytotoxic effects of compounds on SK-N-MC cell line. Cell viability assays using SK-N-MC cells treated with increasing doses of etoposide (10, 15, 20 and 30 µg/ml) and synthesized compounds (0.5, 1, 5, and 10 µg/ml). *P<0.05 compared to DMSO control-treated SK-N-MC cells

The *in vitro* cytotoxic activity of synthesized compounds **4a-e** and **5** was evaluated against three human cancer cell lines including K562 (human erythroleukemia), MDA-MB-231 (human breast cancer), and SK-N-MC (human neuroblastoma) cells (Figures 2, 3, 4). 

In order to determine the concentration required achieving a 50% inhibition of cells induced by each compound, the dose response curve was plotted. The results of cytotoxic assay were mentioned as IC_50_ (µg/ml) of compounds in comparison with standard anti-cancer drug etoposide in Table 1. 

Overall, it is clear that compounds **4a**, **4b**, **4e** and **5** have good cytotoxic activity against tested cancer cell lines. Their IC_50_ values were in the range of 1.85-11.7 µg/ml, while the standard drug etoposide had IC_50_ values between 21.9 and 31.5 µg/ml was less active compared with the mentioned compounds. The activity of the most potent compound **4a** was 6-17 times more than that of etoposide. 

***Acridine orange/ethidium bromide staining test***

**Figure 5:**
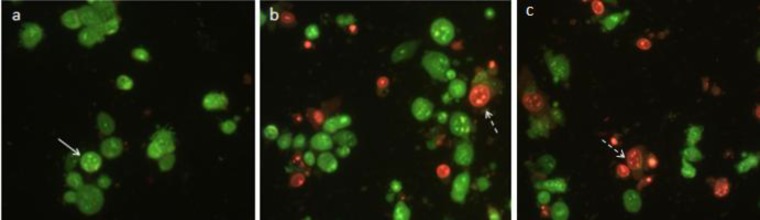
Acridine orange/ethidium bromide fluorescent staining of MDA-MB-231 cells for determine apoptosis: (a) DMSO 1% as control; (b) cells treated with IC_50_ concentration of compound **4a** (c) cells treated with IC_50 _concentration of etoposide as positive control for 24 hr. White arrow indicates live cells and dashed arrow indicates apoptotic cells. The images of cells were taken with a fluorescence microscope at 400×

To determine whether the cytotoxicity of compounds was due to the induction of apoptosis, we assessed the morphology of MDA-MB-231 detached cells in the presence of compound **4a** (the most active compound against MDA-MB-231 cell line) and etoposide with the acridine orange/ethidium bromide staining and fluorescence microscopy. Results showed that the synthetic compound **4a** has growth-inhibitory effects on MDA-MB-231 cells due to the induction of apoptosis. As shown in Figure 5, viable cells showed bright green nucleus due to the staining with acridine orange and also apoptotic cells are stained orange-red by ethidium bromide.

## Discussion

In this study, we have introduced the poly-functionalized 3-benzylidenechroman-4-onesas as new chalcone-like cytotoxic agents. These compounds may be considered as conformationally constrained congeners of chalcones that tolerated poly-functionalization on their core structures for optimization. Although 7-butoxy compound **4b** containing phenolic hydroxy group exhibited good activity against all cell lines (IC_50_ values ≤ 6.2 µg/ mL), other 7-butoxychromanone derivatives **4c** and **4d** showed relatively less or no cytotoxic activity. Thus, it was demonstrated that the *O*-alkyaltion of 4-hydroxybenzylidene moiety diminishes the activity especially against MDA-MB-231 cells. The comparison of the inhibitory activity of 7-hydroxylated compound **4a** and 7-butoxy analogue **4b **revealed that free OH at 7-position of chromanone ring is more favorable. Besides from mixed-functional compounds **4a-d**, the poly-methoxylated compound **4e** exhibited significant growth inhibitory against tested cell lines. The mentioned activities against human erythroleukemia (K562) and human neuroblastoma (SK-N-MC) cells were identical (IC_50_s ≈ 5 µg/ml). Also, this compound was as potent as compound **4b** against K562 and SK-N-MC cell lines. In the present report it was found that, compound **5** which distinguished from other compounds by having 2,3-endocyclic double bond instead of 3-exocyclic double bond showed good cytotoxic activity at least two times better than etoposide. Many of the chalcone-like compounds with various structures have been studied previously for their cytotoxic activities. However, our present understanding of the relationships between their chemical structures and anticancer properties is still very limited. The structural modifications of the chalcones mostly focused on alteration of the phenyl rings along with their substituents (6-9), and conformational restriction of the chalcone to give rigid cyclic analogs (10). A series of 2-benzylidene-1-tetralones **2 **were reported as rigid analogs of chalcones with promising cytotoxic activities against human Molt 4/C8 leukemia, CEM lymphoma, and murine L1210 lymphoma cells (11, 12). Recently, Perjési *et al* designed 3-benzylidenechroman-4-ones **3** as oxygen analogs of 2-benzylidene-1-tetralones **2**. It was shown that a number of 3-benzylidenechroman-4-ones **3 **exhibited greater cytotoxic activity than the corresponding 2-benzylidene-1-tetralones **2** (13). In the current study, we investigated the cytotoxic effect of poly-oxygenated 3-benzylidenechroman-4-ones as new chalcone-like agents. Our results demonstrated that 7-hydroxy group on chromanone ring and 3-bromo-4-hydroxy-5-methoxy on benzylidene is a good pattern for structural optimization of 3-benzylidenechroman-4-one core structure. 

## Conclusion

We described the synthesis and cytotoxic activity of poly-functionalized 3-benzylidenechroman-4-ones** 4** as new chalcone-like agents. These compounds can be considered as conformationally constrained congeners of chalcones that tolerated poly-functionalization on their core structures. The most of compounds showed good inhibitory activity against different cancer cell lines. Among them, compound **4a** containing 7-hydroxy group on chromanone ring and 3-bromo-4-hydroxy-5-methoxy substitution pattern on benzylidene moiety was the most potent compound with IC_50_ values ≤ 3.86 µg/ml. It was 6-17 times more potent than etoposide against tested cell lines. 
